# Stable Isotope Phenotyping via Cluster Analysis of NanoSIMS Data As a Method for Characterizing Distinct Microbial Ecophysiologies and Sulfur-Cycling in the Environment

**DOI:** 10.3389/fmicb.2016.00774

**Published:** 2016-05-26

**Authors:** Katherine S. Dawson, Silvan Scheller, Jesse G. Dillon, Victoria J. Orphan

**Affiliations:** ^1^Division of Geological and Planetary Sciences, California Institute of TechnologyPasadena, CA, USA; ^2^Department of Biological Sciences, California State University Long BeachLong Beach, CA, USA

**Keywords:** NanoSIMS, sulfur-cycling, stable isotope probing, ecophysiology, cluster analysis

## Abstract

Stable isotope probing (SIP) is a valuable tool for gaining insights into ecophysiology and biogeochemical cycling of environmental microbial communities by tracking isotopically labeled compounds into cellular macromolecules as well as into byproducts of respiration. SIP, in conjunction with nanoscale secondary ion mass spectrometry (NanoSIMS), allows for the visualization of isotope incorporation at the single cell level. In this manner, both active cells within a diverse population as well as heterogeneity in metabolism within a homogeneous population can be observed. The ecophysiological implications of these single cell stable isotope measurements are often limited to the taxonomic resolution of paired fluorescence *in situ* hybridization (FISH) microscopy. Here we introduce a taxonomy-independent method using multi-isotope SIP and NanoSIMS for identifying and grouping phenotypically similar microbial cells by their chemical and isotopic fingerprint. This method was applied to SIP experiments in a sulfur-cycling biofilm collected from sulfidic intertidal vents amended with ^13^C-acetate, ^15^N-ammonium, and ^33^S-sulfate. Using a cluster analysis technique based on fuzzy c-means to group cells according to their isotope (^13^C/^12^C, ^15^N/^14^N, and ^33^S/^32^S) and elemental ratio (C/CN and S/CN) profiles, our analysis partitioned ~2200 cellular regions of interest (ROIs) into five distinct groups. These isotope phenotype groupings are reflective of the variation in labeled substrate uptake by cells in a multispecies metabolic network dominated by Gamma- and Deltaproteobacteria. Populations independently grouped by isotope phenotype were subsequently compared with paired FISH data, demonstrating a single coherent deltaproteobacterial cluster and multiple gammaproteobacterial groups, highlighting the distinct ecophysiologies of spatially-associated microbes within the sulfur-cycling biofilm from White Point Beach, CA.

## Introduction

The application of stable isotope probing (SIP) to environmental microbial communities provides links between ecophysiology and phylogenetic identity without the need for pure or enrichment cultures (Radajewski et al., 2000; Dumont and Murrell, 2005). In SIP experiments, a substrate that is enriched in a particular stable isotope (e.g., D, ^13^C, ^15^N, ^34^S) is added to an environmental sample incubation and the uptake of that substrate by members of the microbial community is tracked by the incorporation of the enriched isotope into cellular components. SIP, in combination with fluorescence *in situ* hybridization coupled to secondary ion mass spectrometry (FISH-SIMS or FISH-NanoSIMS; Orphan et al., 2001), can resolve substrate uptake and metabolic activity at the single-cell level within complex communities (Orphan et al., 2009; Wagner, 2009; Musat et al., 2012; Pett-Ridge and Weber, 2012).

SIP combined with FISH-NanoSIMS analysis offers a direct method for assessing the metabolic potential of microorganisms in the environment, where microbial communities are often supported through complex interspecies interactions on the micrometer scale and frequently consist of uncultured and poorly characterized microorganisms. Prior FISH-NanoSIMS studies have focused on single-cell measurements of anabolic activity, metabolic potential, and microbial metabolic interactions particularly with respect to the assimilation of ^13^C-, ^15^N-labeled substrates e.g., (Popa et al., 2007; Musat et al., 2008; Green-Saxena et al., 2014) and recently deuterated water (Berry et al., 2015; Kopf et al., 2015). Very few ecological studies have conducted cell specific SIP experiments with sulfur, despite the fact that sulfur is one of the abundant elements in biomolecules and plays a central role in redox biogeochemistry in many environments. NanoSIMS analyses have previously been applied to measure naturally occurring micron-scale variations in δ^34^S of sulfide resulting from microbial sulfur metabolism in environmental samples (Fike et al., 2008, 2009), and ^34^S-enriched sulfate SIP experiments combined with NanoSIMS have demonstrated the assimilation of ^34^S into cell biomass (Milucka et al., 2012; Wilbanks et al., 2014). These studies focused on the variation in the ratio of ^34^S/^32^S. However, the existence of four stable isotopes of sulfur (^32^S, ^33^S, ^34^S, and ^36^S) and the ability of the CAMECA NanoSIMS 50L instrument to measure seven masses in parallel offers the potential for concurrent SIP NanoSIMS experiments with multiple sulfur species and isotope labels, as well as the potential to conduct mixed substrate incubation experiments that expand beyond ^13^C- and ^15^N-labeled substrate amendment to include multiple isotopes of sulfur.

Inter- and intra-species variation in labeled substrate metabolism associated with differences in growth rates, as well as the transfer of enriched isotope through microbial metabolic networks via cross-feeding of labeled metabolites results in heterogeneity of the isotope ratios measured for different populations (Pelz et al., 1999; Orphan et al., 2001; DeRito et al., 2005; Musat et al., 2008; House et al., 2009; Abraham, 2014; Kopf et al., 2015; Zimmermann et al., 2015). While cross-feeding during SIP incubations is generally considered to be a complicating factor in these experiments (Neufeld et al., 2007; Chen and Murrell, 2010), exploiting the resulting isotopic heterogeneity can move the interpretation of SIP experiments beyond the binary of enriched or not enriched. Using gradients in anabolic activity associated with multiple labeled substrates in combination with cluster analysis has the potential to distinguish metabolic niches, interspecies substrate transfer, and variation due to spatial distribution of microorganisms (DeRito et al., 2005; Chen and Murrell, 2010).

For complex environmental samples, distilling large datasets into manageable groups through clustering techniques supports the generation of hypotheses based on average group properties. Cluster analysis is an exploratory technique that utilizes discontinuities and gradients in multivariate datasets to identify and visualize relationships between subgroups of samples. These groupings can proceed by hierarchical clustering, agglomerating, or dividing samples into clusters and sub-clusters, or partitional methods, where an initial partitioning of the samples is optimized for intra-cluster homogeneity. Both hierarchical and partitional clustering has been applied extensively for identifying relationships within ecological datasets (McCune et al., 2002a,b; Legendre and Legendre, 2012). These techniques have a long history of development and application in macro and meiofaunal community ecology (Green, 1980; James and McCulloch, 1990; Legendre et al., 2005), and have become increasingly common in microbial ecology as applied to DNA or RNA-based diversity data (Ramette, 2007), with an effort toward identifying underlying trends in diversity and links between diversity and function in microbial communities (Fuhrman, 2009). A primary goal of both multivariate and SIP analysis in microbial ecology is identifying connections between identity and functional roles in biogeochemical cycles.

**Figure 1:**
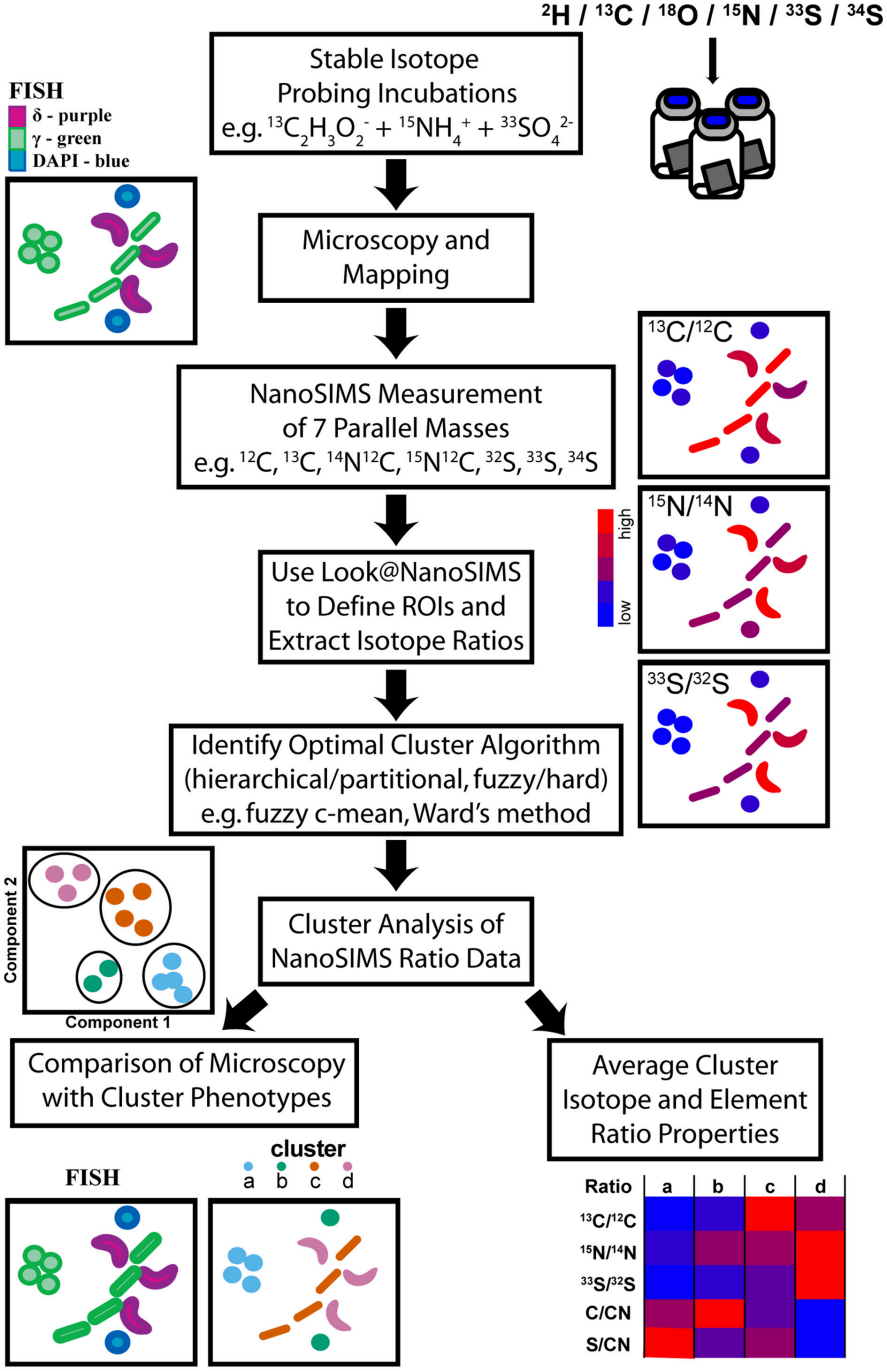
**Illustration of the workflow from multiple-isotope SIP incubations through to the identification of stable isotope phenotypes and their properties via cluster analysis of NanoSIMS data**. After colonization with microbial biomass, NanoSIMS compatible Si-wafers were incubated with multiple stable isotope labeled substrates. Microscopy, using FISH probes, identified regions that were mapped for subsequent NanoSIMS analysis of up to seven parallel masses. NanoSIMS data was processed using Look@NanoSIMS to define single cell regions of interest (ROIs) and extract associated isotope and elemental composition ratios. Several cluster analysis algorithms were evaluated to determine the best method, which was then applied to partition the NanoSIMS ratio data. The properties of the stable isotope phenotype clusters were examined and compared to independent FISH images to investigate label uptake in a multispecies metabolic network.

Here we expand upon the use of cluster analysis as a method to deconvolve multi-isotope NanoSIMS datasets (Figure 1) for microorganisms in environmental samples and describe a case study from a intertidal sulfur cycling, microbial biofilm at White Point Beach, CA after SIP time course incubation with ^13^C, ^15^N, and ^33^S-labeled substrates. Using the high lateral spatial resolution of the NanoSIMS and cluster analysis of ^13^C, ^15^N, and ^33^S enrichment in single cells, we resolved distinct microbial isotopic phenotypes occurring at close spatial scales within a microbial biofilm. Independently, these isotopic phenotypes were found to correlate with distinct delta- and gammaproteobacterial cell types identified by FISH and suggest a microbial, metabolic network for the cycling of carbon and sulfur in chemoautrophic microbial mats associated with sulfidic hydrothermal vents at White Point Beach in San Pedro, CA. The combination of NanoSIMS and cluster analysis in multiple isotope SIP experiments has the potential to provide insights into ecophysiology and element cycling in spatially proximal microorganisms in environmental and laboratory settings.

## Materials and methods

### Case study site description

**Figure 2:**
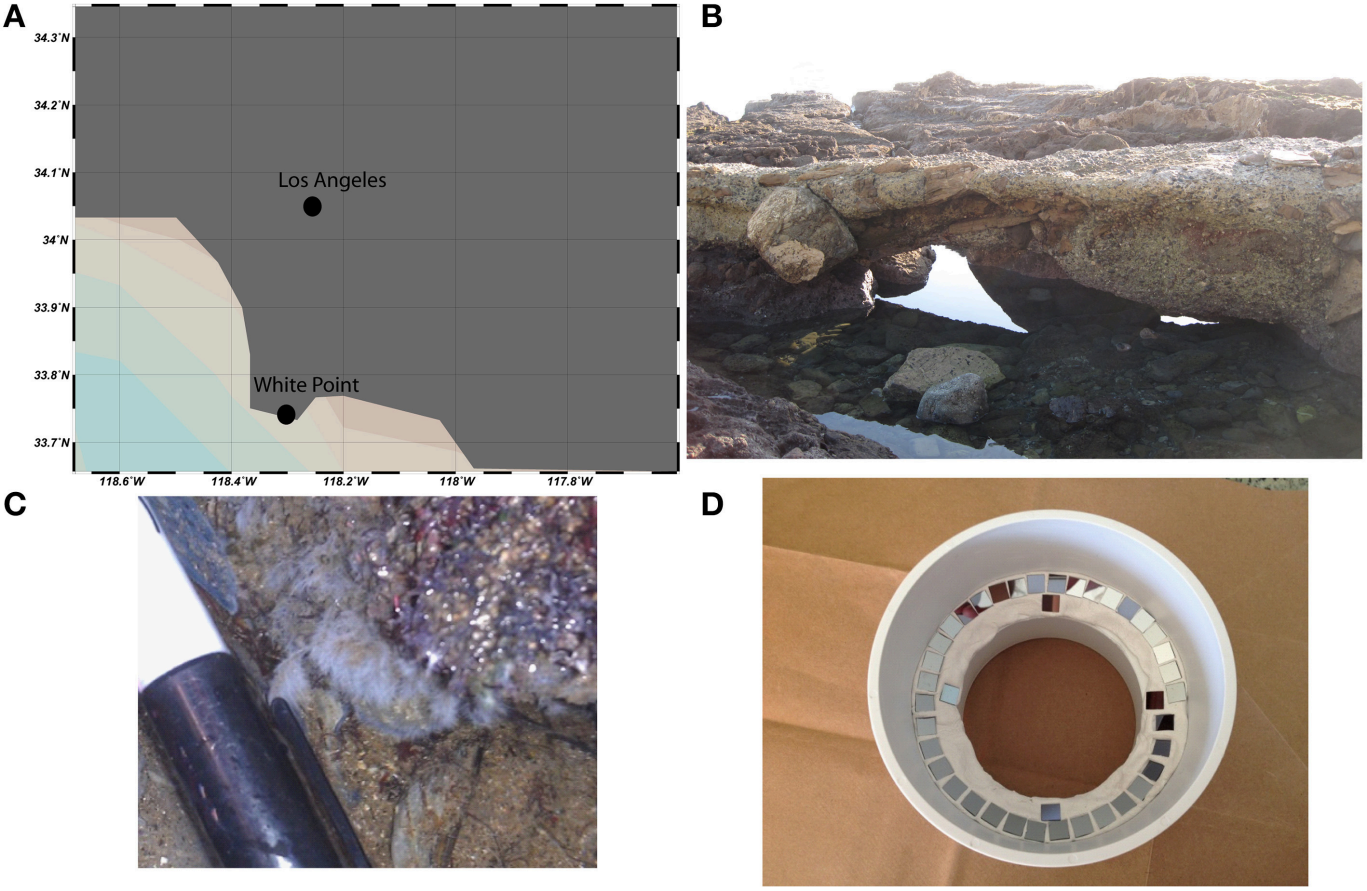
**White Point is located south of Los Angeles, CA on the Palos Verdes Peninsula (A)**. Conspicuous white, filamentous microbial mats form in intertidal pools adjacent to sulfidic hydrothermal vents **(B,C)**. Si-wafers (7 × 7 mm) were colonized *in situ* for 1 week before laboratory incubations with ^13^C-acetate, ^15^${\text{NH}}_{4}^{+}$, and ^34^${\text{SO}}_{4}^{2-}$ **(D)**.

Chemoautrophic microbial mats form in shallow intertidal pools adjacent to sulfidic hydrothermal vents at White Point Beach in San Pedro, CA (33.7159°N, 118.319°W; Stein, 1984; Figure 2). Sulfide is primarily geologically derived from the interaction of hydrothermal fluids with the sulfur containing Altamira shale unit of the Monterrey Formation (Woodring et al., 1946). The location of active venting was identified by enhanced localized colonization of white, filamentous microbial mats on the surface of rocks, sediments, and invertebrates. Illumina tag sequencing of rocks visibly colonized by microbial mat was performed as described previously in Case et al. (2015). Sequencing confirmed prior descriptions of *Thiothrix* spp. as the dominant sulfur-oxidizing bacteria in the microbial mats (Stein, 1984; Jacq et al., 1989; Kalanetra et al., 2004). Additional gammaproteobacterial groups belonging to the Thiotrichaceae and Oceanospirillaceae families accounted for 95% of the Gammaproteobacteria (35.8 and 4.1% relative abundance, respectively). Sequencing additionally identified putative sulfur- and sulfate-reducing deltaproteobacterial lineages belonging to the Desulfuromonadaceae, Desulfobacteraceae, and Desulfobulbaceae families (1.75, 0.51, and 0.22% relative abundance, respectively). The raw sequences from the *in situ* White Point mats were generated on an Illumina MiSeq platform at Laragen, Inc (Los Angeles, CA, USA) and have been deposited in the Sequence Read Archive (accession # PRJNA304767).

### Triple isotope probing experiments

Microbial mat biomass for the SIP experiments was collected by *in situ* colonization of NanoSIMS compatible contact slides. Specifically, 7 × 7 mm conductive Si-wafers (P-type/boron doped, 0.028 Ω cm^−1^, 725 μm thick, Active Business Company Gmbh, Munich, Germany) were secured to a slide holder and incubated for 1 week in the intertidal pool adjacent to locations of active sulfide venting (Figure 2). The microbial mat colonized wafers were then transferred into 10 ml serum bottles containing 5 ml of filter-sterilized, N_2_-sparged, sulfate-free artificial sea water that contained (g l^−1^): 24 g NaCl; 5 g ${\text{MgCl}}_{2}^{.}$6H_2_O; 1.31 g ${\text{CaCl}}_{2}^{.}$2H_2_O; 0.67 g KCl; 0.2 g NaHCO_3_; 0.1 g KBr; 0.027 g H_3_BO_3_; 0.027 g ${\text{SrCl}}_{2}^{.}$6H_2_O; 0.003 g NaF. Incubations were then amended with ^13^C-acetate, ^15^${\text{NH}}_{4}^{+}$, ^33^${\text{SO}}_{4}^{2-}$, 20% air/80% N_2_ headspace, with each treatment consisting of three parallel bottles with two replicate Si-wafers per bottle. After 2, 7, and 10 days one bottle per treatment was sacrificed and sampled for geochemistry, microscopy, and NanoSIMS analyses.

Additional incubations included an unlabeled control (NL) and a formaldehyde killed control (K), which were supplemented as follows: NL—acetate, ${\text{NH}}_{4}^{+}$, ${\text{SO}}_{4}^{2-}$, 20% air/80% N_2_ headspace; K—^13^C-acetate, ^15^${\text{NH}}_{4}^{+}$, ^15^${\text{NO}}_{3}^{-}$, ^33^${\text{SO}}_{4}^{2-}$, 10% w/v formaldehyde, 20% air/80% N_2_ headspace. Final concentrations and atom percent (at.%) enrichment for the supplements added to all additional incubations were as follows: sodium acetate—100 μM, 2-^13^C, 99 at.% ^13^C (Cambridge Isotope Laboratories, Tewksbury, MA, USA); NH_4_Cl—100 μM, ~10 at.% ^15^N (Cambridge Isotope Laboratories, Tewksbury, MA, USA); NaNO_3_—100 μM, 98% ^15^N (Cambridge Isotope Laboratories, Tewksbury, MA, USA); Na_2_SO_4_—28 mM, ~15 at.% ^33^S (see below for synthesis details). The at.% ^15^N and ^33^S added to incubations was calculated by isotope mass balance as follows: ^n^F_final_ = [(^n^F_unlabeled_× m_unlabeled_) + (^n^F_labeled_× m_labeled_)]/m_final_, where mass (m) was the amount of NH_4_Cl or NaSO_4_ added to the mixture and at.% = 100 × ^n^F.

### Synthesis of ^33^S-sulfate from ^33^S-elemental sulfur

Na_2_SO_4_ enriched in ^33^S was prepared in house by the oxidation of ^33^S^0^ (99.8%, Trace Sciences International Inc., Wilmington, DE, USA). The oxidation reaction was conducted in a custom apparatus constructed from 6 mm glass tubing (Figure S-1). ^33^S^0^ powder (170 mg) was placed into the glass tube and briefly melted to adhere to the glass with a bunsen burner. A gentle stream of pure O_2_ (Air Liquide, USA) was passed through the glass tube (ca. 20 ml min^−1^) and the other side was immersed into a 0.4 M NaOH solution (10 ml). After passing several volumes of O_2_ through the apparatus, the sulfur was ignited in the tube by heating the outside with an ethanol-flame. The liquid sulfur burned with a blue flame and the resulting SO_2_ was quantitatively absorbed in the NaOH solution. Glass wool had been placed behind the burning sulfur to prevent S^0^ vapors from reaching the NaOH solution. Once reacted the solution of ${\text{Na}}_{2}^{33}$SO_3_ (10 ml) was oxidized with an excess of 10 M H_2_O_2_ (500 μl) and vortexed. This reaction occurred within 5 min and complete oxidation was assumed by the presence of residual H_2_O_2_ using MQuant peroxide-test strips (EMD Millipore, Temecula, CA, USA). The excess H_2_O_2_ was decomposed by incubating the solution at 90°C for 20 h. Decomposition of all H_2_O_2_ was again confirmed with peroxide-test strips. The pH of the solution was adjusted to 4.0 by titration with 6 M HCl in 100 μl increments. The acidic solution was then filtered through a 0.22 μm filter, and neutralized with 1 M NaOH. Conversion to ${\text{SO}}_{4}^{2-}$ was verified and quantified by ion chromatography using a Dionex ICS-2000 system with an IonPac AS18 anion exchange column (Dionex, Sunnyvale, CA, USA). Yields were typically >90% and ${\text{SO}}_{3}^{2-}$ was not detected.

### Fluorescence *in situ* hybridization

At 2, 7, and 10 days the microbially colonized Si-wafers from one bottle of each experimental condition were gently washed with 3X phosphate buffered saline (PBS; g l^−1^: 24 g NaCl, 0.6 g KH_2_PO_4_, 0.6 g KCl, 4.32 g Na_2_HPO_4_) to remove residual labeled media and fixed for microscopy by adding 4% fresh paraformaldehyde and 3x PBS in a 3:1 ratio and incubating at 4°C for 12 h. Fixed samples were subsequently washed with 3x PBS and stored in 3x PBS at 4°C. Ethanol was avoided to prevent the dissolution of internal sulfur granules.

Prior to FISH, the Si-wafers were etched with a series of grid lines using a LMD7000 laser microdissection system (Leica, Wetzlar, Germany) to provide a map for identifying sample locations on the NanoSIMS. FISH was performed as described previously (Glöckner et al., 1996), using fluorescently labeled oligonucleotide probes targeting Gammaproteobacteria (Gam42a; Manz et al., 1992) and Deltaproteobacteria (Delta495a + cDelta495a; Loy et al., 2002; Macalady et al., 2006). Oligonucleotide probes were labeled at the 5′ end with either a FAM or CY3 dye (IDT, Coralville, IA USA) and used at a final concentration of 5 ng μl^−1^ in hybridization buffer containing 45% formamide. Following the FISH hybridization, the cells were counterstained with 4′,6-diamidino-2-phenylindole (DAPI) at a final concentration of 2.5 ng μl^−1^ and coverslips were mounted with the anti-fade reagent, Vectashield (Vector Laboratories, USA). Regions positively hybridized with the FISH probes near the etched grid lines were mapped and imaged on a BX51 epifluorescence microscope (Olympus, Shinjuku, Japan) using 20x (UPlanFL N) dry, 60x (PlanApo N) and 100x (UPlanFL N) oil immersion objectives. After FISH analysis, coverslips were gently removed from the Si-wafers to minimize dislodging the cells and the water-soluble Vectashield was washed away with MQ water in a petri dish as described in Dekas and Orphan (2011).

### ^13^C, ^15^N, and ^33^S analysis of single cells using FISH-NanoSIMS

Carbon, nitrogen, and sulfur isotopic compositions of microbial cells from mapped regions were measured using a NanoSIMS 50L (CAMECA, Gennevilliers, France) housed in the Center for Microanalysis in the Division of Geological and Planetary Sciences at the California Institute of Technology. All measurements included in this study were made in one continuous 10-day session on the instrument. Cells were analyzed on Si-wafers using a ~4 pA primary Cs^+^ beam current and were pre-sputtered with a ~16 pA primary Cs^+^ beam current for 10–15 min. Seven masses were collected in parallel (^12^C^−^, ^13^C^−^, ^14^N^12^C^−^, ^15^N^12^C^−^, ^32^S^−^, ^33^S^−^, and ^34^S^−^). Secondary ion images were collected for 30 × 30 μm raster areas at 512 × 512 pixel resolution with a dwell time of 15 ms/pixel for 20–40 cycles. *Clostridia* spores of a known isotopic composition (measured by EA-IRMS, δ^13^C = −21.86%0; ^13^R = 0.01099 and δ^15^N = 7.94%0; ^15^R = 0.00371) were analyzed daily to correct for instrumental isotope fractionation in the ^13^C/^12^C and ^15^N/^14^N ratios and to assess any instrument drift over the course of the run (Dekas and Orphan, 2011). No drift was detected over the 10-day period. To determine the instrumental isotope fractionation by the NanoSIMS instrument, the average NanoSIMS acquired ^13^C/^12^C and ^15^N/^14^N ratios were compared with the EA-IRMS acquired values for the *Clostridia* spores. A correction for ^33^S/^32^S was not possible due to the low sulfur content in the spores. However, it is unlikely that this relatively minor correction (permil) would alter the results of this analysis given the large atom percent enrichments observed in the cells after incubation with the labeled substrates. Raw data from all secondary ion images were processed using Look@NanoSIMS in MATLAB (Polerecky et al., 2012). Ion images were corrected for dead time, planes were accumulated and aligned, and discrete regions of interest (ROIs, *n* = 3115) were drawn by hand using the ^14^N^12^C^−^ ion image to identify and outline individual cells.

### Cluster analysis

Accumulated secondary ion counts, exported from Look@NanoSIMS, were used to calculate isotopic ratios for ^13^C/^12^C, ^15^N/^14^N (^15^N^12^C/^14^N^12^C), ^33^S/^32^S, and ^34^S/^32^S. Additionally, ratios of C/CN and S/CN were calculated from the sum of accumulated secondary ion counts for C (^12^C + ^13^C), CN (^14^N^12^C + ^15^N^12^C), and S (^32^S + ^33^S + ^34^S). The isotope and element composition ratios were compiled for individual raster areas as well as all raster areas corresponding to a given incubation time point. Cluster analysis was performed in the R environment (RStudio v.0.99.451, R v.3.2.1; R Core Team, 2015; R Studio Team, 2015) using clustering algorithms from the “cluster” package (Maechler et al., 2015), cluster method and number validation from the “clusterSim” (Walesiak et al., 2008) and “clusterCrit” (Desgraupes, 2015) packages, respectively, and graphical output from the “ggplot2” package (Wickham, 2009). Using “clusterSim” and “clusterCrit,” 10 cluster analysis methods [single-linkage, median-linkage, average-linkage, complete-linkage, centroid-linkage, Ward's method, McQuitty's method, partitioning around medoids (pam), k-means and fuzzy c-means] were compared using the Calinski-Harabasz (CH) validity index (Caliński and Harabasz, 1974) for solutions where the number of clusters ranged between 2 and 10. Scripts written for processing data associated with this project can be found at https://github.com/katdawson/NanoSIMS-cluster-analysis.

The two main cluster analysis methods used to interpret the NanoSIMS isotope and elemental ratio datasets were k-means and fuzzy c-means. K-means cluster analysis starts with a fixed number of clusters (k) and starting configuration of cluster centers. The analysis proceeds by assigning objects, in this case ROIs, to clusters and iteratively optimizing the object memberships such that the sum of square distance is minimized between objects and the cluster center (Lance and Williams, 1967; MacQueen, 1967). Fuzzy c-means cluster analysis similarly requires an initial number of clusters to which objects are assigned, but allows objects to belong to more than one cluster. Fuzzy c-means iteratively optimizes both the cluster center and memberships (Bezdek et al., 1984; Equihua, 1990).

## Results

### Fish-NanoSIMS analysis of cellular C, N, and S

**Figure 3:**
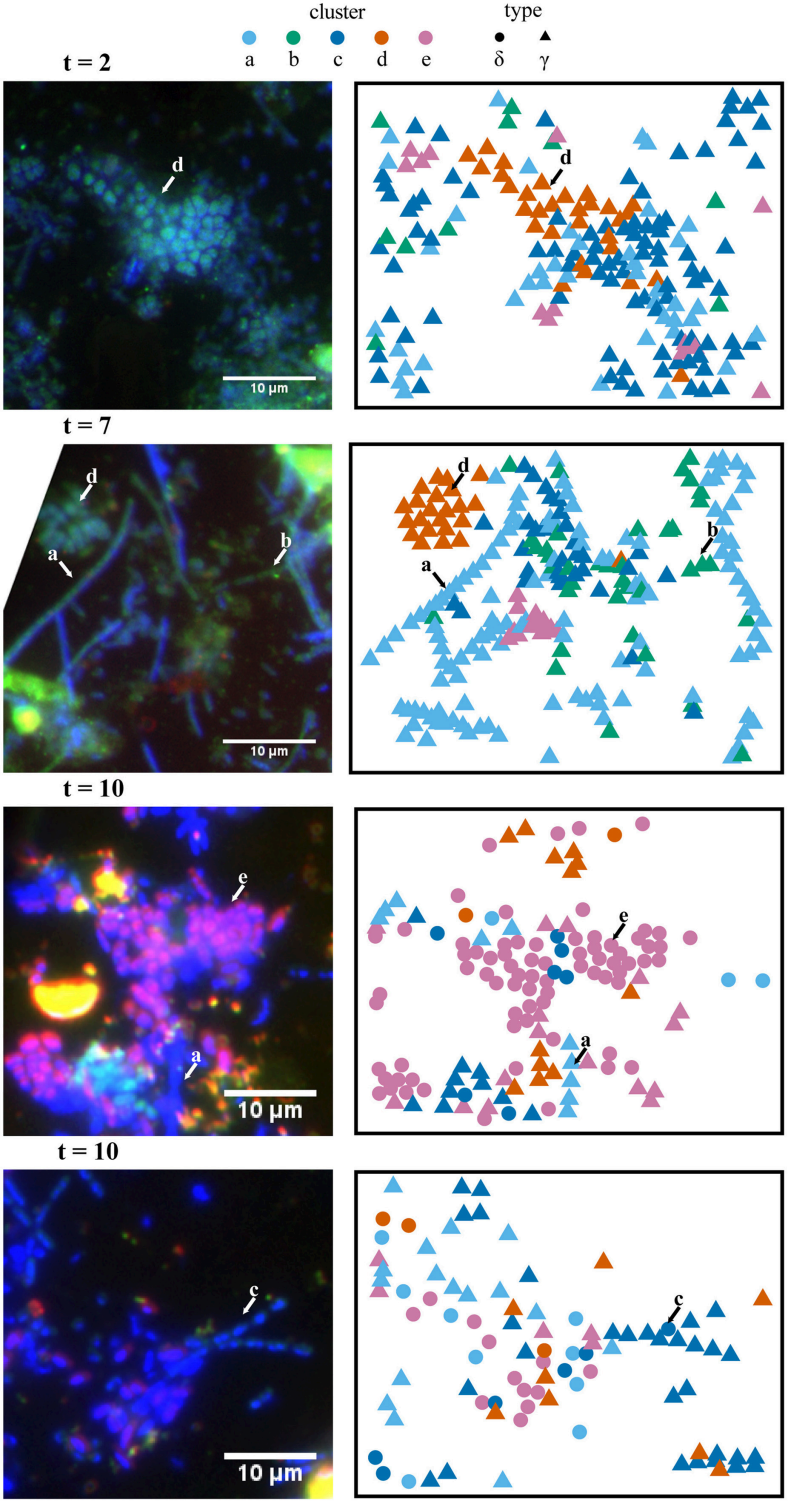
**Spatial (x,y) projections of isotope phenotype clusters determined from NanoSIMS data and corresponding FISH images**. Independent analysis of these two data sets showed consistent correspondence between the isotope phenotypes (clusters a–e) and the morphologies and phylogenetic affiliation of cells determined in the FISH experiments. For example, cluster “e” primarily corresponds with putative sulfate-reducing bacteria hybridized with a deltaproteobacterial probe (purple). FISH (left panels) was performed with probe Delta495a targeting Deltaproteobacteria (purple) and Gam42a targeting Gammaproteobacteria (green). Representative cells for isotope phenotype clusters a–e, determined from fuzzy c-means cluster analysis of NanoSIMS data, are indicated with arrows in the FISH images. Clusters “a,” “b,” and “c” are primarily affiliated with filamentous Gammaproteobacteria. Cluster “d” is primarily affiliated with clusters of coccoidal Gammaproteobacteria, andcluster “e” is primarily affiliated with Deltaproteobacteria. FISH images were rotated to match the NanoSIMS image orientation, which resulted in the cropping of some images.

FISH analyses of Si-wafers for the labeled incubation time series (2, 7, and 10 days) as well as unlabeled (7 day) and killed (10 day) control incubations were carried out using oligonucleotide probes targeting Gammaproteobacteria (Gam42a) and Deltaproteobacteria (Delta495a + cDelta495a; Figure 3). The relative abundance of Deltaproteobacteria increased over the course of the experiment: 2 days—3.0%; 7 days—6.8%; 10 days—44.7%. Deltaproteobacteria represented 3.6% of imaged cells in the unlabeled control and 11.9% of imaged cells in the killed control. These differences highlight the inherent heterogeneity of microbial assemblage composition during the *in situ* colonization in the intertidal vents at White Point.

A total of 21 FISH mapped regions (30 × 30 μm) on five incubated Si-wafers were examined by NanoSIMS and subsequently processed using Look@NanoSIMS resulting in 3115 cellular ROIs (Table 1, Figure S-4). These mapped areas included three regions from the 2-day labeled incubation (665 ROIs), four regions from the 7-day labeled incubation (777 ROIs), six regions from the 7-day labeled incubation (760 ROIs), three regions from the unlabeled control (645 ROIs), and three regions from the killed control (268 ROIs). Isotope ratios for ROIs from the different time points of the ^13^C, ^15^N, and ^33^S-labeled substrate incubations had a broad distribution, with the standard deviations of similar magnitude to the mean ratios (Table 1). Over the 10-day incubation period with ^15^${\text{NH}}_{4}^{+}$, the mean ^15^N/^14^N for ROIs identified as Deltaproteobacteria by FISH increased from 0.0447 ± 0.0169 at day 2 to 0.0628 ± 0.0203 by day 10 (ROIs in unlabeled control = 0.00347 ± 0.00019). In incubations amended with ^33^${\text{SO}}_{4}^{2-}$, the mean ^33^S/^32^S for deltaproteobacterial cells increased from 0.0187 ± 0.0065 (day 2) to 0.1292 ± 0.1224 (day 7), followed by a decrease to 0.0578 ± 0.0430 at day 10. Gammaproteobacterial ROIs in those same incubations had an average ^15^N/^14^N ratio of 0.0354 ± 0.0125 which did not change appreciably during the 10 day incubation. However, FISH-identified gammaproteobacterial cells did show an increase in ^33^S/^32^S from 0.0138 ± 0.0106 at day 2 to 0.0302 ± 0.0270 by day 10. Amendments with ^13^C-acetate produced similar trends in ^13^C/^12^C for both delta- and gammaproteobacterial cells with the peak in ^13^C enrichment observed early in the incubation series at day 2 (0.1432 ± 0.0915) followed by a decrease to 0.0984 ± 0.0335 at day 7, with cells in the final incubation bottle (day 10) showing slightly a slightly higher ^13^C/^12^C ratio (0.1040 ± 0.0649). Cells recovered from the unlabeled control incubation did not show any isotope enrichment (^13^C/^12^C—0.0109 ± 0.00051; ^15^N/^14^N—0.00347 ± 0.00019; ^33^S/^32^S—0.00720 ± 0.00076) and only minor enrichment was detected for ^13^C/^12^C and ^15^N/^14^N in cells from the killed control amended with the same suite of isotopically enriched substrates (^13^C/^12^C—0.0123 ± 0.0032; ^15^N/^14^N—0.0045 ± 0.00066; ^33^S/^32^S—0.00568 ± 0.0011). Previous SIMS studies have reported similar levels of isotopic enrichment in cells above natural abundance from killed control experiments amended with isotopically labeled substrates (e.g., ^15^${\text{NH}}_{4}^{+}$; Orphan et al., 2009; Kopf et al., 2015).

**Table 1:** **Average isotope and elemental ratio properties for all cellular ROIs, the FISH-identified Delta- and Gammaproteobacteria, and the five cluster, fuzzy c-means solutions for all incubation and control experiments**.

	**All**	**Delta**	**Gamma**	**a**	**b**	**c**	**d**	**e**
**2 DAYS:**^13^**C-ACETATE** + ^15^${\text{NH}}_{4}^{+}$ + ^33^${\text{SO}}_{4}^{2-}$								
Number of ROIs	665	20	645	253	107	257	30	18
^13^C/^12^C	0.1432	0.1817	0.1421	0.0625	0.2284	0.1889	0.1086	0.1757
sd	0.0915	0.1098	0.0915	0.0363	0.1024	0.0580	0.0486	0.0974
^12^C^15^N/^12^C^14^N	0.0394	0.0447	0.0393	0.0187	0.0569	0.0508	0.0370	0.0704
sd	0.0216	0.0169	0.0216	0.0112	0.0158	0.0123	0.0167	0.0259
^33^S/^32^S	0.0139	0.0187	0.0138	0.0094	0.0164	0.0141	0.0122	0.0622
sd	0.0106	0.0065	0.0106	0.0028	0.0070	0.0056	0.0051	0.0245
C/CN	0.4080	0.4286	0.4073	0.4239	0.5943	0.3383	0.2667	0.3066
sd	0.1405	0.1219	0.1405	0.1159	0.1267	0.0830	0.0842	0.1191
S/CN	0.1187	0.1032	0.1192	0.1084	0.1415	0.1028	0.2554	0.1281
sd	0.0452	0.0222	0.0452	0.0219	0.0213	0.0206	0.1106	0.0268
**7 DAYS:**^13^**C-ACETATE** + ^15^${\text{NH}}_{4}^{+}$ + ^33^${\text{SO}}_{4}^{2-}$								
Number of ROIs	777	53	724	443	136	106	32	60
^13^C/^12^C	0.0984	0.0727	0.0760	0.0304	0.0499	0.2687	0.0694	0.1323
sd	0.0335	0.0506	0.1009	0.0298	0.0385	0.1112	0.0397	0.0850
^12^C^15^N/^12^C^14^N	0.0283	0.0688	0.0214	0.0105	0.0157	0.0532	0.0217	0.1001
sd	0.0309	0.0471	0.0235	0.0105	0.0100	0.0125	0.0103	0.0162
^33^S/^32^S	0.0620	0.1292	0.0259	0.0151	0.0150	0.0306	0.0078	0.2229
sd	0.4579	0.1224	0.0481	0.0109	0.0102	0.0249	0.0016	0.0899
C/CN	0.2067	0.2726	0.4715	0.3736	0.7612	0.5187	0.4769	0.2759
sd	0.1113	0.1608	0.2032	0.1042	0.1953	0.1529	0.2435	0.1203
S/CN	0.0564	0.0880	0.1130	0.0886	0.1509	0.1188	0.3125	0.0690
sd	0.0593	0.0873	0.0531	0.0193	0.0326	0.0275	0.0815	0.0193
**10 DAYS:**^13^**C-ACETATE** + ^15^${\text{NH}}_{4}^{+}$ + ^33^${\text{SO}}_{4}^{2-}$								
Number of ROIs	760	340	420	306	0	158	66	230
^13^C/^12^C	0.1040	0.0938	0.1123	0.0655	n.d.	0.1943	0.0910	0.0968
sd	0.0649	0.0487	0.0746	0.0385	n.d	0.0638	0.0452	0.0307
^12^C^15^N/^12^C^14^N	0.0531	0.0628	0.0454	0.0379	n.d.	0.0561	0.0448	0.0739
sd	0.0222	0.0203	0.0207	0.0207	n.d	0.0104	0.0196	0.0106
^33^S/^32^S	0.0425	0.0578	0.0302	0.0146	n.d.	0.0285	0.0446	0.0887
sd	0.0377	0.0430	0.0270	0.0169	n.d.	0.0233	0.0283	0.0199
C/CN	0.4303	0.3493	0.4960	0.3113	n.d.	0.5350	0.8700	0.3907
sd	0.2528	0.1787	0.2832	0.1853	n.d.	0.1987	0.3895	0.1100
S/CN	0.0792	0.0804	0.0783	0.0648	n.d.	0.0650	0.1660	0.0833
sd	0.0436	0.0370	0.0482	0.0243	n.d.	0.0134	0.0905	0.0213
**UNLABELED CONTROL (7 DAYS): ACETATE** + ${\text{NH}}_{4}^{+}$ + ${\text{SO}}_{4}^{2-}$								
Number of ROIs	645	23	622	155	133	39	193	125
^13^C/^12^C	0.0109	0.0109	0.0109	0.0109	0.0105	0.0106	0.0109	0.0112
sd	0.0005	0.0004	0.0005	0.0003	0.0003	0.0004	0.0003	0.0003
^12^C^15^N/^12^C^14^N	0.0035	0.0035	0.0035	0.0036	0.0034	0.0034	0.0034	0.0034
sd	0.0002	0.0001	0.0002	0.0001	0.0001	0.0001	0.0001	0.0001
^33^S/^32^S	0.0072	0.0074	0.0072	0.0074	0.0074	0.0092	0.0068	0.0067
sd	0.0008	0.0005	0.0008	0.0004	0.0004	0.0009	0.0003	0.0002
C/CN	0.3362	0.4385	0.3324	0.3687	0.4739	0.4149	0.1868	0.3557
sd	0.1507	0.1484	0.1507	0.0891	0.1442	0.1209	0.0701	0.1202
S/CN	0.1101	0.1167	0.1099	0.0656	0.0697	0.0652	0.1103	0.2220
sd	0.0691	0.0847	0.0691	0.0168	0.0274	0.0213	0.0387	0.0607
**KILLED CONTROL (10 DAYS):** ^13^**C-ACETATE** + ^15^${\text{NH}}_{4}^{+}$ + ^15^${\text{NO}}_{3}^{-}$+ ^33^${\text{SO}}_{4}^{2-}$								
Number of ROIs	268	32	236	110	79	55	13	11
^13^C/^12^C	0.0123	0.0143	0.0120	0.0115	0.0123	0.0116	0.0120	0.0241
sd	0.0032	0.0027	0.0032	0.0015	0.0015	0.0010	0.0009	0.0060
^12^C^15^N/^12^C^14^N	0.0045	0.0046	0.0045	0.0046	0.0041	0.0044	0.0064	0.0048
sd	0.0007	0.0004	0.0007	0.0004	0.0003	0.0003	0.0008	0.0005
^33^S/^32^S	0.0057	0.0056	0.0057	0.0049	0.0063	0.0061	0.0066	0.0061
sd	0.0011	0.0011	0.0011	0.0009	0.0007	0.0008	0.0015	0.0003
C/CN	0.3114	0.3128	0.3112	0.3165	0.1999	0.4473	0.2845	0.4123
sd	0.1325	0.0370	0.1325	0.0729	0.0935	0.1433	0.0504	0.0903
S/CN	0.0917	0.0800	0.0933	0.0738	0.0855	0.1433	0.0684	0.0841
sd	0.0429	0.0373	0.0429	0.0137	0.0255	0.0618	0.0287	0.0225

*Gamma, Gammaproteobacteria; Delta, Deltaproteobacteria; sd, standard deviation; n.d., not detected*.

### Cluster method and number validation

**Figure 4:**
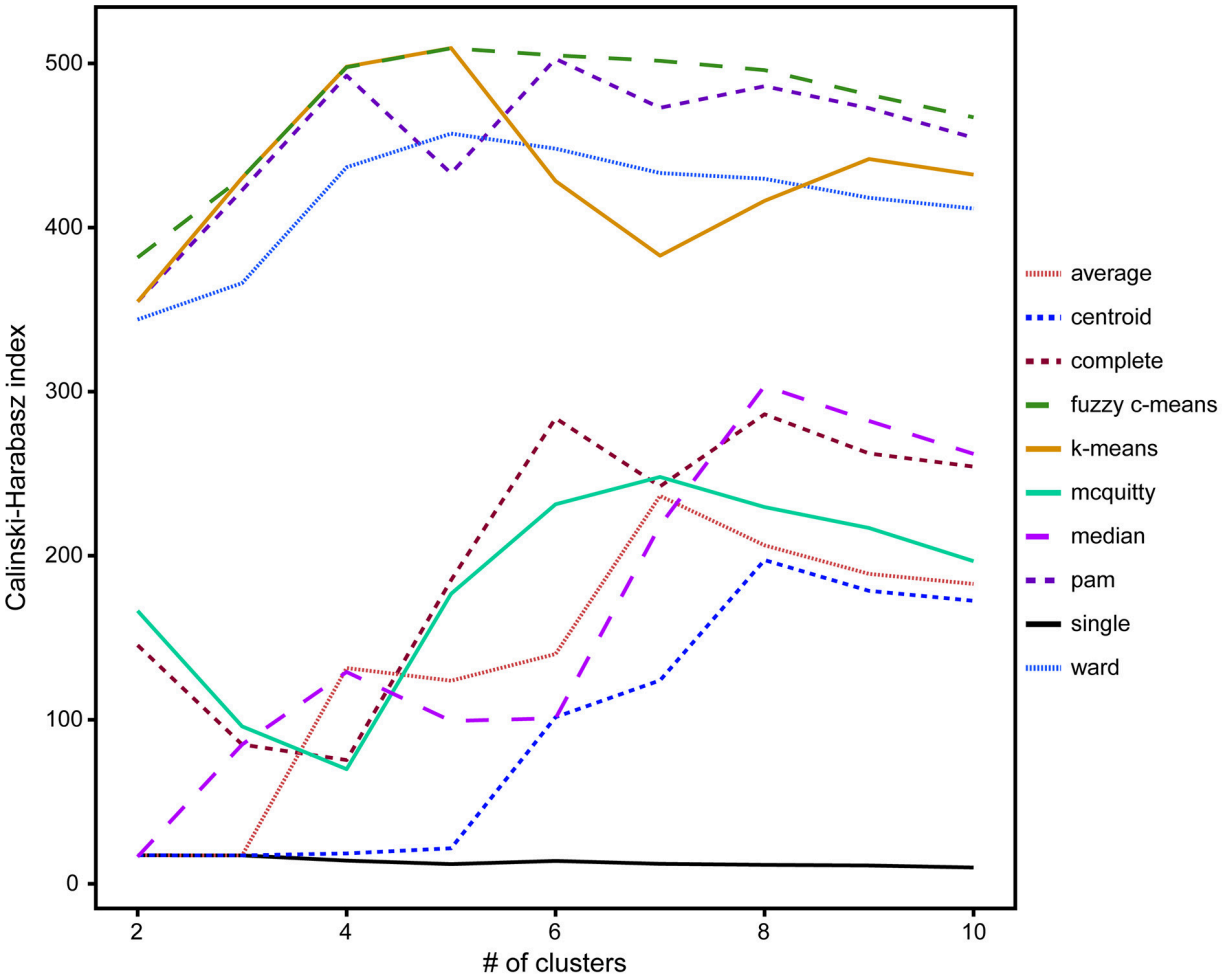
**A comparison of the 10 clustering algorithms with the Calinski-Harabasz validity index (CH index) for solutions containing between 2 and 10 clusters, where higher index values indicated better intra-cluster cohesion and inter-cluster separation**. Of these clustering methods, the partitional methods fuzzy c-means (dashed green) and k-means (solid orange) with five clusters were found to be the optimal algorithms for the isotope and elemental ratio NanoSIMS data.

A comparison of hierarchical (single-linkage, median-linkage, average-linkage, complete-linkage, centroid-linkage, Ward's method, and McQuitty's method) and partitional (pam, k-means and fuzzy c-means) cluster analysis methods using the CH validity index revealed that partitional algorithms better optimized groups of ROIs by isotope phenotype. These methods rely on cluster ellipsoids drawn to minimize the variance between the member ROIs and the mean cluster value, resulting in the best representation of normal distributions anticipated from biological activity (Figure 4; Legendre and Legendre, 2012). Higher CH index values represent better intra-cluster cohesion and inter-cluster separation (Caliński and Harabasz, 1974). With the exception of the single-linkage method, five to eight clusters maximized the CH index. The highest CH index values were determined for the following partitional methods, fuzzy c-means (5 clusters, CH = 509.2), k-means (5 clusters, CH = 509.4), pam (6 clusters, CH = 502.8), and Ward's method (5 clusters, CH = 457.3; Figure 4).

**Figure 5:**
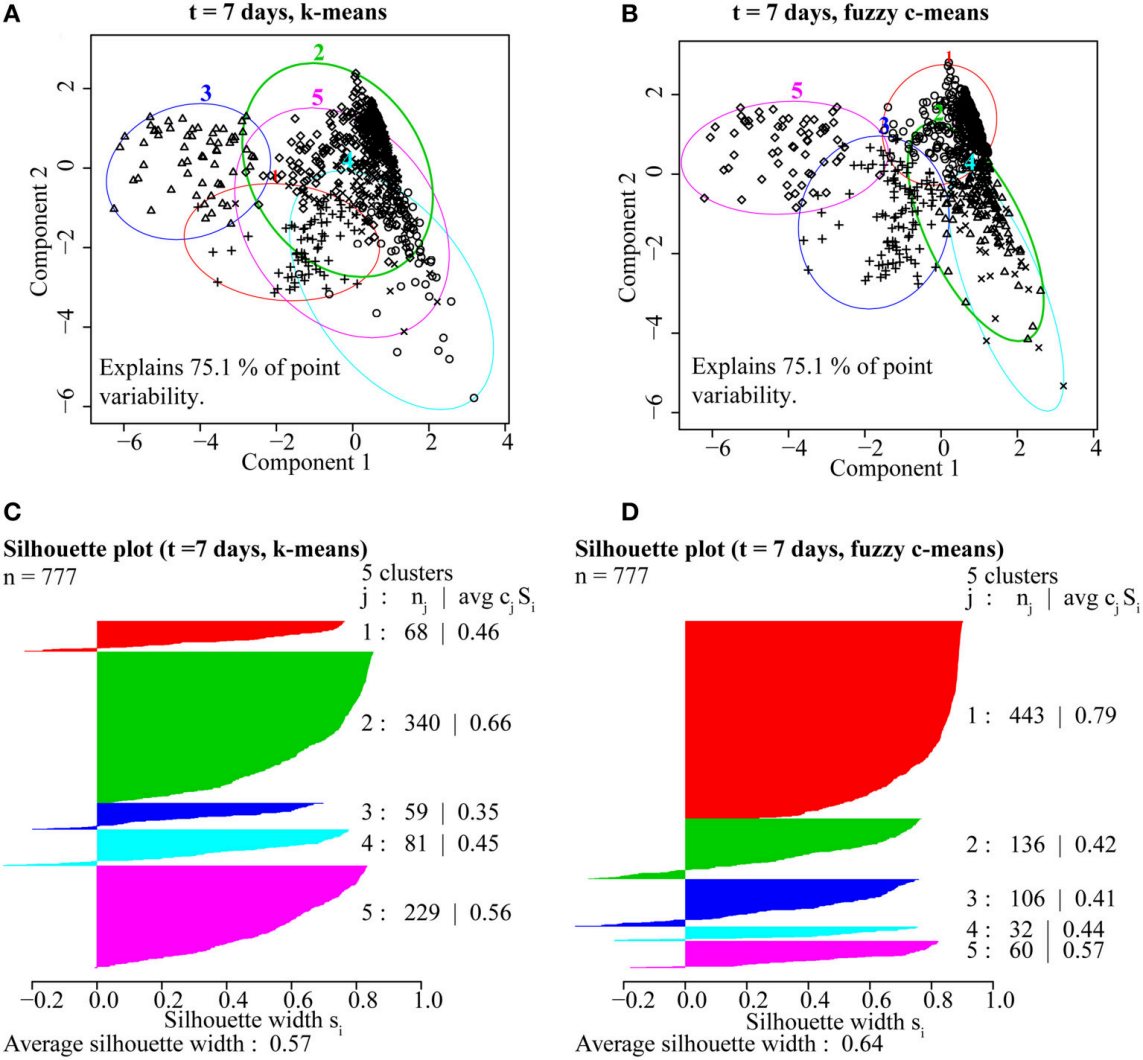
**A comparison of partitioning by (A) k-means and (B) fuzzy c-means for ROIs from the ***t*** = 7 day NanoSIMS isotope and elemental ratio data showed that fuzzy c-means resulted in better cluster resolution**. The fuzzy c-means clustering solution minimized overlap **(A,B)** and resulted in higher average silhouette width (avg c_j_S_j_), a measure of intra-cluster cohesion and inter-cluster separation, for ROIs (n_j_) distributed into the five clusters (j; **C,D)**.

Based on these findings (Figure 4), k-means and fuzzy c-means clustering with five clusters appear to be equally valid methods for clustering NanoSIMS isotope ratio data and were used for all subsequent data analysis. Further comparison of k-means and fuzzy c-means, showed less visual overlap in the fuzzy c-means clusters projected into vector space (Figure 5B). Greater cluster differentiation with fuzzy c-means was confirmed by increased average and overall cluster silhouette width (Figure 5D). Silhouette plots provide a graphical assessment of cluster solutions, by combining the inter-cluster separation and the intra-cluster cohesion into a width for each object that increases with improvement in both parameters (Rousseeuw, 1987). The average silhouette widths indicated that clustering by fuzzy c-means improved partitioning of the data compared to the k-means method. However, there was still substantial overlap in the clusters defined by fuzzy c-means, particularly between clusters 1, 2, and 4. This overlap (Figure 5) was visible in both the projection of the clusters in bivariate plots and by the presence of negative silhouette widths.

### Test of isotope phenotyping with 1, 2, or 3 isotope ratios

In order to determine the benefit of additional ratio data for determining isotope phenotypes, we compared the five cluster fuzzy c-means assignments resulting from isotope and elemental ratio data derived from one (^15^N/^14^N), two (^15^N/^14^N, ^13^C/^12^C, C/CN), or three (^15^N/^14^N, ^13^C/^12^C, ^33^S/^32^S, C/CN, S/CN) labeled substrates. These assignments were examined by projecting ROIs into x,y coordinate space alongside the corresponding FISH image (Figure 4, Table 2). In this comparative analysis, some ROI assignments remained consistent across all cluster analysis solutions (*n* = 171, 22.0%), and the addition of the ^13^C/^12^C and ^33^S/^32^S ratios improved the differentiation of non-filamentous cell types. The incorporation of all three isotope ratios (^15^N/^14^N,^13^C/^12^C, and ^33^S/^32^S) resulted in the best correspondence between the NanoSIMS acquired cellular data and FISH image, revealing potential discrepancies in phylogenetic assignment of some ROIs identified by FISH. For these cells, the FISH-based assignment tended to be less clear, compromised by inherent autofluorescence in the sample, variability in fluorescence intensity between the larger filamentous microorganisms and co-occurring single cells, or weak hybridization possibly due to poor specificity of the oligonucleotide probe.

**Table 2:** **Cluster assignments for a subset of ROIs (***n*** = 777) using isotope and elemental ratios resulting from 1, 2, or 3 stable isotope labeled substrates**.

**Ratios used**	**FISH identification**	**Distribution of ROIs per cluster**				
		**a**	**b**	**c**	**d**	**e**
^13^C/^12^C	Gamma	416	142	77	52	38
	Delta	13	27	12	1	0
^15^N/^14^N	Gamma	385	125	96	120	32
	Delta	13	2	6	5	27
^33^S/^32^S	Gamma	579	81	24	33	7
	Delta	18	5	1	17	12
^13^C/^12^C, ^15^N/^14^N, C/CN	Gamma	309	77	207	82	51
	Delta	16	1	4	0	32
^13^C/^12^C, ^33^S/^32^S, C/S	Gamma	483	100	29	84	29
	Delta	10	21	2	1	19
^15^N/^14^N, ^33^S/^32^S, S/CN	Gamma	428	226	35	27	8
	Delta	14	8	2	16	13
^13^C/^12^C, ^15^N/^14^N, ^33^S/^32^S, C/CN, C/SN	Gamma	427	134	101	30	32
	Delta	16	2	5	2	28

*Gamma, Gammaproteobacteria; Delta, Deltaproteobacteria*.

### Cluster assignment consistency and properties

Projection of the ROI x,y coordinate data for various raster areas and incubation time points (Figure 3) showed consistency between the cluster assignment and the independent taxon identification by FISH microscopy. Filamentous Gammaproteobacteria (putative *Thiothrix* spp.) were primarily associated with clusters “a,” “b,” and “c”; 86, 98, and 92% of the ROIs were FISH identified as Gammaproteobacteria, respectively. Cluster “d” was associated with non-filamentous cells consisting of both FISH-identified Gammaproteobacteria (85% of ROIs), as well as cells stained by DAPI with an undetermined phylogenetic affiliation (15% of ROIs). Deltaproteobacteria were primarily associated with cluster “e,” with 68% of ROIs hybridizing with the deltaproteobacterial specific FISH probe, Delta495a.

Cross-plots of the ROI isotope and elemental ratios (Figure 5) demonstrated that clusters are associated with specific properties (Table 1). For example, cluster “a” ROIs had the lowest ratios of ^13^C/^12^C, ^15^N/^14^N, and ^33^S/^32^S, while the cluster “b” ROIs were also characterized by low ^13^C/^12^C, ^15^N/^14^N, and ^33^S/^32^S, but had higher elemental C/CN and S/CN ratios compared to other groups. ROIs in cluster “c” had the highest ^13^C/^12^C, with moderate ^15^N/^14^N, and low ^33^S/^32^S. Cluster “d” ROIs were similar to cluster “a” and “b” in terms of low isotopic ratios, but had the greatest (S/CN)/(C/CN) ratio. ROIs in cluster “e” were characterized by the highest ^15^N/^14^N and ^33^S/^32^S, with low to moderate ^13^C/^12^C and low C/CN.

Natural variation in the stable isotope and elemental ratios recorded for cells in the unlabeled and killed control samples did not contain large enough gradients for the cluster algorithm to differentiate different cell types, despite the inherent variation in elemental ratios observed for some cell populations (Figures S-2, S-3). Cross-plots of isotope ratios showed minimal uptake of the ^13^C and ^15^N label in the killed control and a distribution of ROIs within one standard deviation of the calculated natural abundance value for all ratios in the unlabeled control. In both killed and unlabeled control samples, cross-plots of elemental ratios identified a subset of cell ROIs with higher S/CN consistent with the elevated ratios observed from a subset of the ROIs in the SIP experiments (Figure 5, cluster “d”).

## Discussion

Since the development of FISH-SIMS and FISH-NanoSIMS for directly measuring the metabolic activity of taxonomically identified single microbial cells in environmental samples (Orphan et al., 2001; Wagner, 2009) this methodological approach has gained widespread use in the field of microbial ecology, illuminating within and between population differences in anabolic activity and ecophysiology of environmental microorganisms (House et al., 2009; Musat et al., 2012; Green-Saxena et al., 2014; Zimmermann et al., 2015). To extend the utility of this approach, we developed a taxonomy independent method that combines cluster analysis with multi-isotope SIP and NanoSIMS for identifying and grouping microbial cells with phenotypically similar isotopic ratios and chemical compositions reflective of distinct ecophysiologies (Figure 1). Cluster analysis utilizes discontinuities and gradients in multivariate datasets to identify and visualize relationships between groupings, here represented by combinations of isotopic enrichment or elemental ratios for environmental microorganisms after SIP. The distillation of complex datasets into defined groupings facilitates data interpretation and new hypothesis development based on average group properties and composition. Similar clustering approaches, including the fuzzy–c-means algorithm, have been applied in the field of environmental metagenomics, enabling the binning of sequence reads within complex data sets (e.g., Nasser et al., 2008; Liu et al., 2015; Lu et al., 2016).

Here we show that clustering algorithms can be applied to NanoSIMS-acquired isotopic and elemental ratio data sets of single cells recovered from SIP experiments, enabling the identification of ecophysiological characteristics and trends for co-existing microorganisms and microbial populations in environmental samples. There are a number of different clustering algorithms available for characterizing multivariate datasets. Comparison of 10 different clustering algorithms, generally divided into either partitional or hierarchical-based clustering methods, revealed that partitional methods yielded more robust cluster solutions for our NanoSIMS isotope and element ratio data set, identifying five clusters (Figure 4). The success of partitional methods here is likely related to the underlying structure of our data, which consisted of gradients in isotopic enrichment resulting from anabolic activity and cross-feeding within the microbial community.

This methodological approach was then tested in a low complexity, sulfur cycling microbial mat community recovered from a hydrothermally influenced intertidal area at White Point beach, CA. NanoSIMS analysis of multi-isotope SIP experiments yielded ~2200 single cell measurements of ^13^C/^12^C, ^15^N/^14^N, and ^33^S/^32^S, as well as the associated cellular elemental ratios C/CN and S/CN. Fuzzy c-means cluster analysis partitioned data into five isotope phenotypes that were reflective of differences in the anabolism of acetate, sulfate, and ammonium. Comparison of these isotope phenotypes, which were determined solely by multivariate analysis of quantitative isotope and elemental ratio data, to corresponding FISH images supported the efficacy of cluster assignments to particular phylogenetic groups, and demonstrated the variability in general anabolic activity and the metabolism of carbon and sulfur within taxonomically related cells.

**Figure 6:**
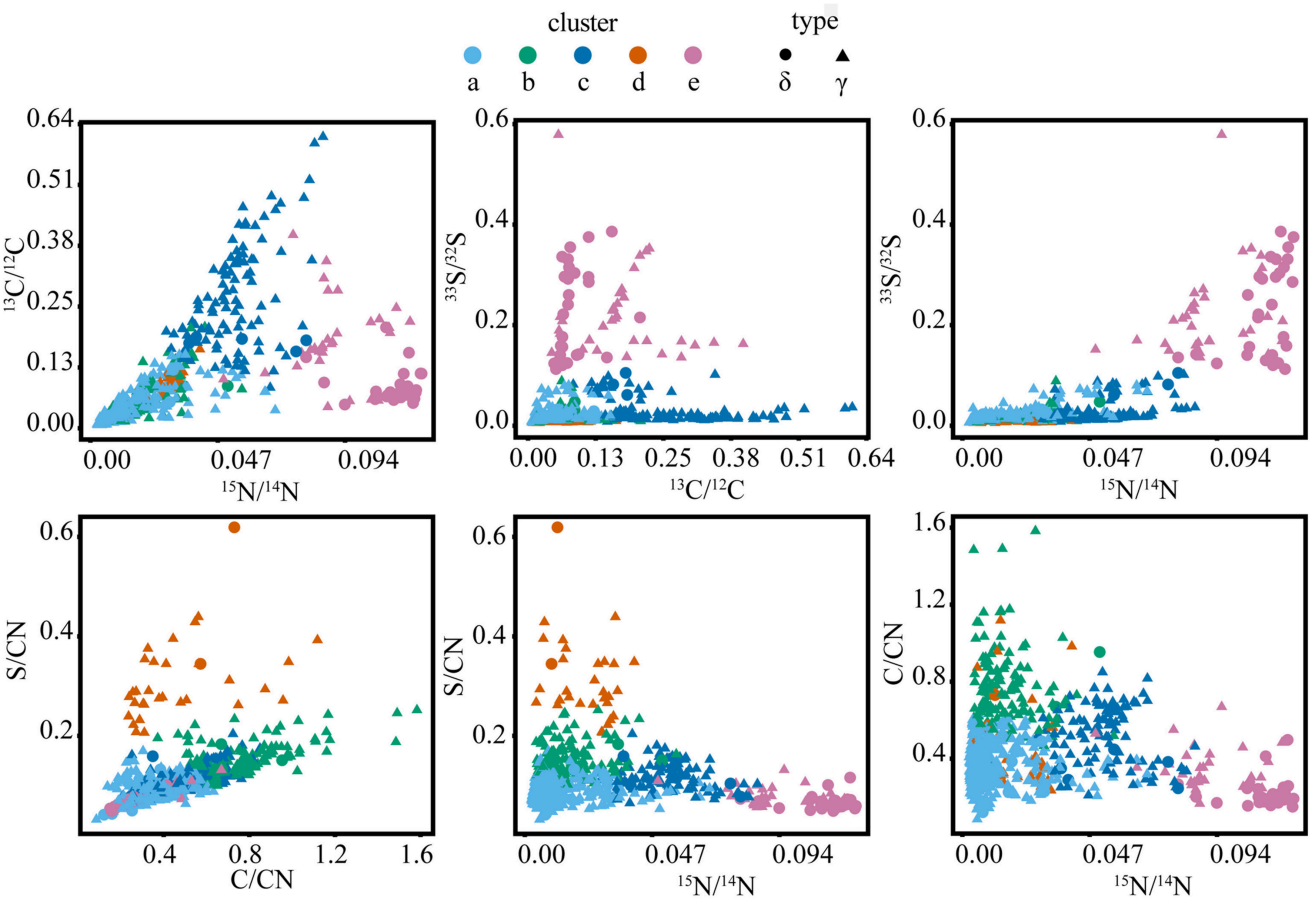
**Isotope and elemental ratio properties of the five clusters from the full SIP dataset (***t*** = 2, 7, and 10 days), containing a total of 2202 cellular ROIs, which were used to determine isotope phenotypes from the sulfur cycling White Point microbial assemblage**. Clusters “a,” “b,” and “c” are primarily affiliated with filamentous Gammaproteobacteria (“γ”). Cluster “d” is primarily affiliated with clusters of coccoid Gammaproteobacteria. Cluster “e” is primarily affiliated with Deltaproteobacteria (“δ”).

**Figure 7:**
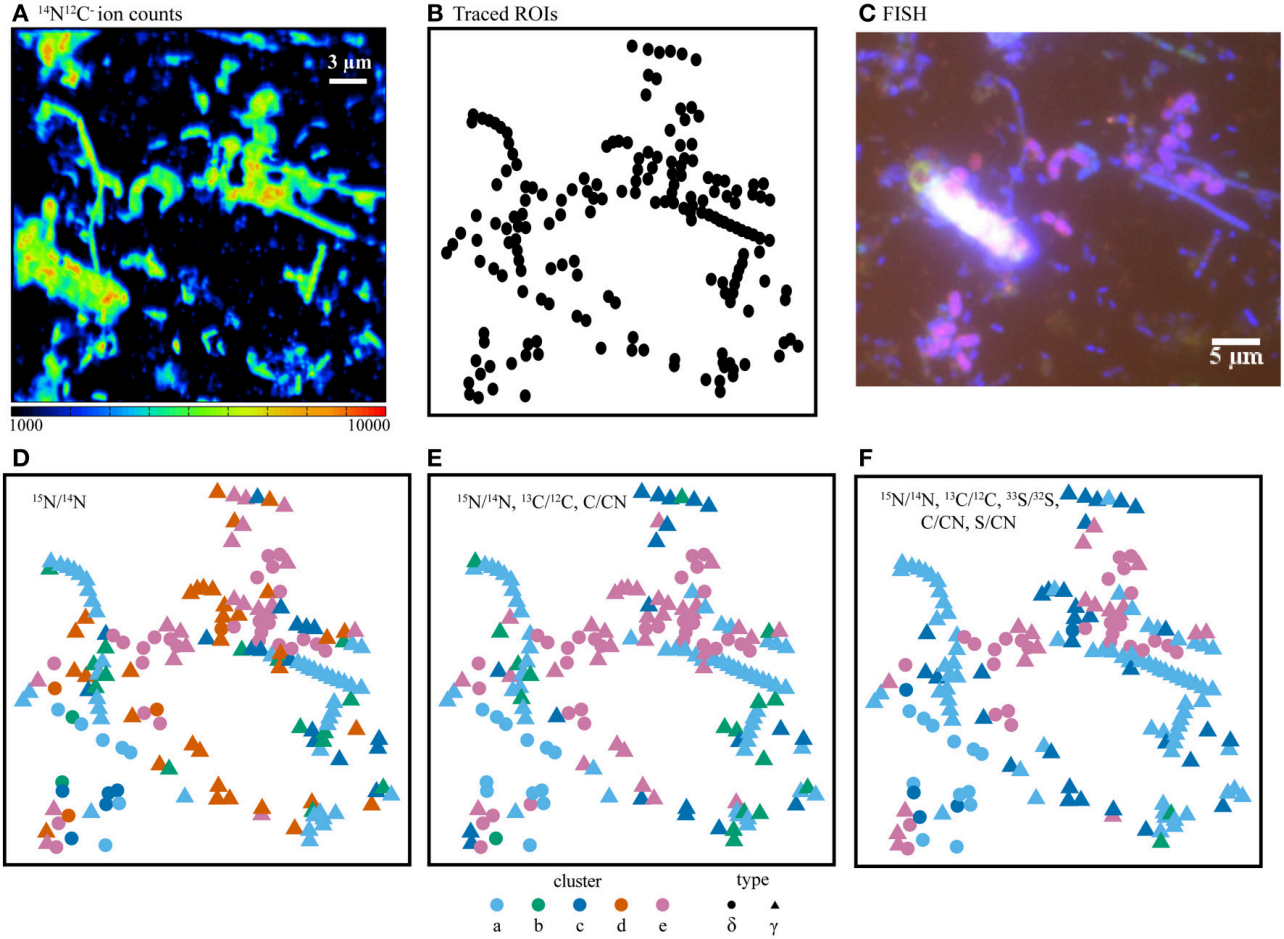
**(A)** NanoSIMS ion counts from ^14^N^12^C^−^ as a proxy for biomass, **(B)** cell outlines (ROIs), and **(C)** the corresponding FISH image. Comparison of isotope and elemental ratio data resulting from SIP experiments using ^15^${\text{NH}}_{4}^{+}$ + ^13^C-acetate + ^33^${\text{SO}}_{4}^{2-}$ where fuzzy c-means cluster determination was based on **(D)** one ratio, **(E)** three ratios, or **(F)** five ratios. Here, the use of five ratios provided the best differentiation of cell types as compared with the independent FISH data. In panels **D–F**, ROI colors were determined by cluster assignment (a–e). Phylogenetic assignments to “δ” (Deltaproteobacteria) or “γ” (Gammaproteobacteria) were determined by overlaying ROI positions in the NanoSIMS and FISH images prior to cluster analysis. FISH experiments **(C)** were performed with probes for Deltaproteobacteria (purple), Gammaproteobacteria (blue-green), and counter-stained with DAPI (blue).

While many SIP and isotope tracer experiments utilize one or two isotope labels (Radajewski et al., 2000; Dumont and Murrell, 2005; Wegener et al., 2012), the Cameca NanoSIMS 50L instrument has the capability of measuring seven masses in parallel, providing a means for tracing the flow of three or four independently labeled substrates in an experiment. The incubations reported here used ^15^${\text{NH}}_{4}^{+}$, ^13^C-acetate, and ^33^${\text{SO}}_{4}^{2-}$ to investigate N, C, and S cycling. The benefit of multiple stable isotope amendments for identifying metabolic niches in incubation samples was explored by applying the different clustering algorithms with data from one, two, or three different isotope labels (Figure 6). In all cases, clusters of cells were identified on the basis of trends in isotope ratios derived from metabolism of the labeled substrates. The use of a single ratio, in this case ^15^N/^14^N, served as a general activity-based proxy, providing one dimension of information related to ^15^${\text{NH}}_{4}^{+}$ assimilation and protein synthesis (e.g., Orphan et al., 2009; McGlynn et al., 2015). The addition of ^13^C/^12^C from ^13^C-acetate and the elemental ratio C/CN added additional dimensions to the cluster analysis, potentially related to differences in intracellular carbon storage and the metabolism of ^13^C-labeled acetate or ^13^C-labeled dissolved inorganic carbon derived from acetate catabolism. A third dimension for discrimination of phenotypic cell types was gained through the addition of ^33^S/^32^S and S/CN. The sulfur isotope and elemental ratios partitioned cells based on ^33^S-sulfate reduction, subsequent oxidation of ^33^S-sulfide, and intracellular sulfur storage. Gradients in the ^13^C/^12^C, ^15^N/^14^N, and ^33^S/^32^S ratios emphasized variation in carbon, nitrogen, and sulfur metabolism within the mat microbial community and resulted in better resolution of different phenotypic cell types (Figures 3, 7; Figure S-4).

Cluster analysis partitioned ~2200 ROIs from a total of 13 raster areas, distributed across three independent Si-wafers representing different SIP incubation time points (2, 7, and 10 days). Cluster assignment and cell type determined by FISH for the corresponding epifluorescence images was consistent for multiple mapped regions on the same Si-wafer and for mapped regions on independent Si-wafers at all-time points (Figures 3A, 7; Figure S-4). The observed correlation between cluster and cell type across these different samples suggest that the isotope and element ratios of ROIs are coherent and likely reflective of distinct ecophysiologies or metabolic niches in this system. Filamentous, sulfide-oxidizing Gammaproteobacteria (“a,” “b,” and “c”) and sulfate-reducing Deltaproteobacteria (“e”), which represented the metabolic endmembers of sulfur cycling in this system, stood out for their robustness. Additionally, two clusters (“a” and “e”), showed the lowest intra-cluster variance and greatest inter-cluster separation (Figure 5) indicating that the combined isotope and elemental ratios better differentiated these ROIs (Figure 6).

The defining features of the five microbial phenotypes were captured in a series of isotope and element ratio biplots (Figure 6). Three of these phenotypes emerged as particularly distinct both in their isotope and elemental ratios (“c,” “d,” and “e”). Cluster “c” consisted of the primary acetate metabolizers (highest ^13^C/^12^C) and included a subset of cells that may be the principal sulfide oxidizers (second highest ^33^S/^32^S). These phenotypic properties were consistent with FISH analysis, which indicated that cluster “c” was primarily composed of filamentous, sulfide-oxidizing Gammaproteobacteria. These Gammaproteobacteria are likely *Thiothrix* spp., identified by iTAG sequencing and previously reported to incorporate acetate (Nielsen et al., 2000). Based on comparison to FISH images, clusters “a,” “b,” and “c” were morphologically similar filamentous Gammaproteobacteria, which likely reflected a gradient of low (“a” and “b”) to high (“c”) anabolic activity (^15^N/^14^N) and acetate utilization. An activity gradient is further supported by a predominance of filaments with the less active “a” and “b” phenotypes in earlier time points (2 and 7 days), while later time point filaments showed an increase in the more active “c” phenotype and the absence of the “b” phenotype in the 10-day incubation (Figures 3, 6). Cluster “b” is differentiated further by high ratios of C/CN, which may indicate the presence of carbon storage granules (Larkin and Strohl, 1983; Rossetti et al., 2003). In cultured *Thiothrix* relatives, intracellular carbon storage compounds were found to increase during acetate metabolism (Rossetti et al., 2003). However, none of these groups displayed high S/CN ratios, suggesting an absence of sulfur storage globules in the incubated cells despite their observation in samples analyzed immediately after field collection. The lack of sulfur storage granules in the cells analyzed from the SIP time course experiments may be attributed to the low concentrations of sulfide, thiosulfate, and nitrate measured in incubations (data not shown), as well as the transition from aerobic to anaerobic conditions by the end of the incubation period. These conditions may have prompted these bacteria to oxidize sulfur stores generated during non-limiting conditions associated with colonization in the White Point intertidal pools (Nielsen et al., 2000; Okabe et al., 2005; Dahl and Prange, 2006).

Similar to cluster “a,” the ROIs of cluster “d” showed lower ^15^${\text{NH}}_{4}^{+}$ incorporation and ^13^C-acetate derived carbon assimilation when compared to cluster “c.” In contrast to the FISH-identified filamentous Gammaproteobacteria associated with clusters “a,” “b,” and “c,” the non-filamentous Gammaproteobacteria associated with cluster “d” exclusively included ROIs with high amounts of sulfur to biomass (high S/CN ratio), possibly indicative of the presence of sulfur storage globules (Figure 6). A portion of the cluster “d” phenotype also showed high amounts of carbon to biomass (high C/CN), which may additionally indicate carbon storage granules. Comparison to FISH images showed that cluster “d” was associated with aggregates of coccoid Gammaproteobacteria, as well as other gammaproteobacterial cells and unidentified (DAPI-stained) single rods (Figure 3). Given the elevated S/CN ratio for this cluster, it is possible that these cells may also be sulfur-metabolizing microorganisms not targeted by our FISH probes (e.g., sulfur-oxidizing, Epsilonproteobacteria affiliated with *Sulfurovum* recovered in the White Point iTAG sequencing data).

Cluster “e” ROIs contained the primary sulfate reducers (highest ^33^S/^32^S), which were also the most anabolically active cells (highest ^15^N/^14^N; Figure 6). While 68% of cells within cluster “e” were FISH-identified as Deltaproteobacteria, an additional 32% of cells were only stained by DAPI and were not hybridized by either the Delta495a or GAM42a FISH probes (Figure 3, Figure S-4). The latter group may represent another sulfate-reducing bacterial group not targeted by the Delta495a probe, or perhaps are due to suboptimal FISH hybridization. The unidentified members of cluster “e” showed greater acetate-derived carbon assimilation (high ^13^C/^12^C), but lower ^15^${\text{NH}}_{4}^{+}$ uptake than their FISH-hybridized, deltaproteobacterial counterparts. Neither subset of cluster “e” displayed high ratios of S/CN or C/CN, which indicated that these cells did not contain sulfur or carbon storage granules. The co-occurrence of more than one group of sulfate- or sulfur-reducing bacteria within cluster “e” is further supported by the presence of multiple families of Deltaproteobacteria (Desulfuromonadaceae, Desulfobacteraceae, and Desulfobulbaceae), as well as a putative thiosulfate- and sulfur-reducing *Fusibacter* spp. also identified in the iTAG data. These findings correspond with a companion study that reported low, but detectable rates of sulfate-reduction and identified members of the sulfur-reducing genus *Desulfuromusa* in the White Point mats (Miranda et al., in review).

Resolution of different cell types in these experiments relied on the combined metabolism of C, N, and S stable isotope labeled substrates to emphasize interspecies variation in three different element cycling pathways. Using the seven different collectors on the NanoSIMS 50L instrument additionally enables expansion of this method to include up to three distinct isotopically labeled sulfur substrates, including ^33^S, ^34^S, and/or ^36^S. The ability to label independently both oxidized and reduced sulfur pools in the same incubation may offer additional insights into the sulfur cycling processes occurring within the White Point microbial mat community, as well as in other sulfur-based ecosystems. Alternatively, triple isotope SIP experiments utilizing ${\text{H}}_{2}^{18}$O or D_2_O as tracers for cellular growth and activity (Schwartz, 2007; Berry et al., 2015; Kopf et al., 2015) may also be directly combined with various ^13^C and ^15^N labeled substrates to expand the scope of the methods demonstrated here with sulfur metabolizing systems to ones where carbon and nitrogen metabolism drive the ecological dynamics. In this sulfur-cycling case study, we identified fuzzy c-means as the optimal clustering algorithm. While fuzzy c-means is likely to work well in other biological systems, testing and comparison of multiple clustering techniques is recommended as this technique is expanded to other environments and different multiple-isotope SIP combinations.

The major benefits of the triple isotope SIP experiments coupled to cluster analysis method described here are the microscopy-independent classification of microbial cells based on their phenotypic properties. Variability in the signal intensity of FISH probes, competing background autofluorescence, and taxonomic specificity are inherent problems for epifluorescence microscopy investigations of microorganisms in complex environmental systems, often complicating data interpretation and limiting the identification of subpopulations. This method offers a unique approach to quantitatively identify subpopulations of cells based on multivariate analysis of gradients in the assimilation of multiple isotopically labeled substrates and, if applicable, differences in elemental stoichiometry. These gradients in labeled substrate metabolism and anabolic activity may reflect ecophysiological niches, and thus the isotope phenotype groupings provide a taxonomically independent means of assessing metabolic networks within environmental microbial communities.

## Author contributions

KD designed and carried out experiments and wrote the manuscript. VO designed experiments and wrote the manuscript. SS developed the labeled sulfur oxidation protocol and provided feedback on the manuscript. JD provided valuable contextual information for experimental design and feedback on the manuscript.

### Conflict of interest statement

The authors declare that the research was conducted in the absence of any commercial or financial relationships that could be construed as a potential conflict of interest.
