# Supplementary material for: Stable Isotope Phenotyping via Cluster Analysis of NanoSIMS Data As a Method for Characterizing Distinct Microbial Ecophysiologies and Sulfur-Cycling in the Environment
Source: Front Microbiol. 2016 May 26;7:774. doi: 10.3389/fmicb.2016.00774 (PMC4881376; doi:10.3389/fmicb.2016.00774)
Supplement: Supplementary file 1 [file Presentation1.PDF]

Supplemental Material

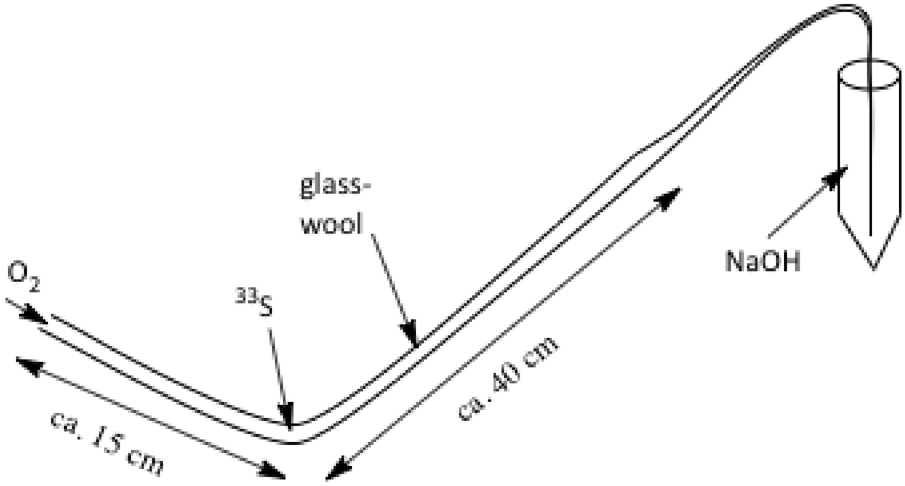

Figure S-1: Diagram of the apparatus used to oxidize elemental sulfur in the synthesis of ^{33}S-SO_4^{2-} from $^{33}S^0$.

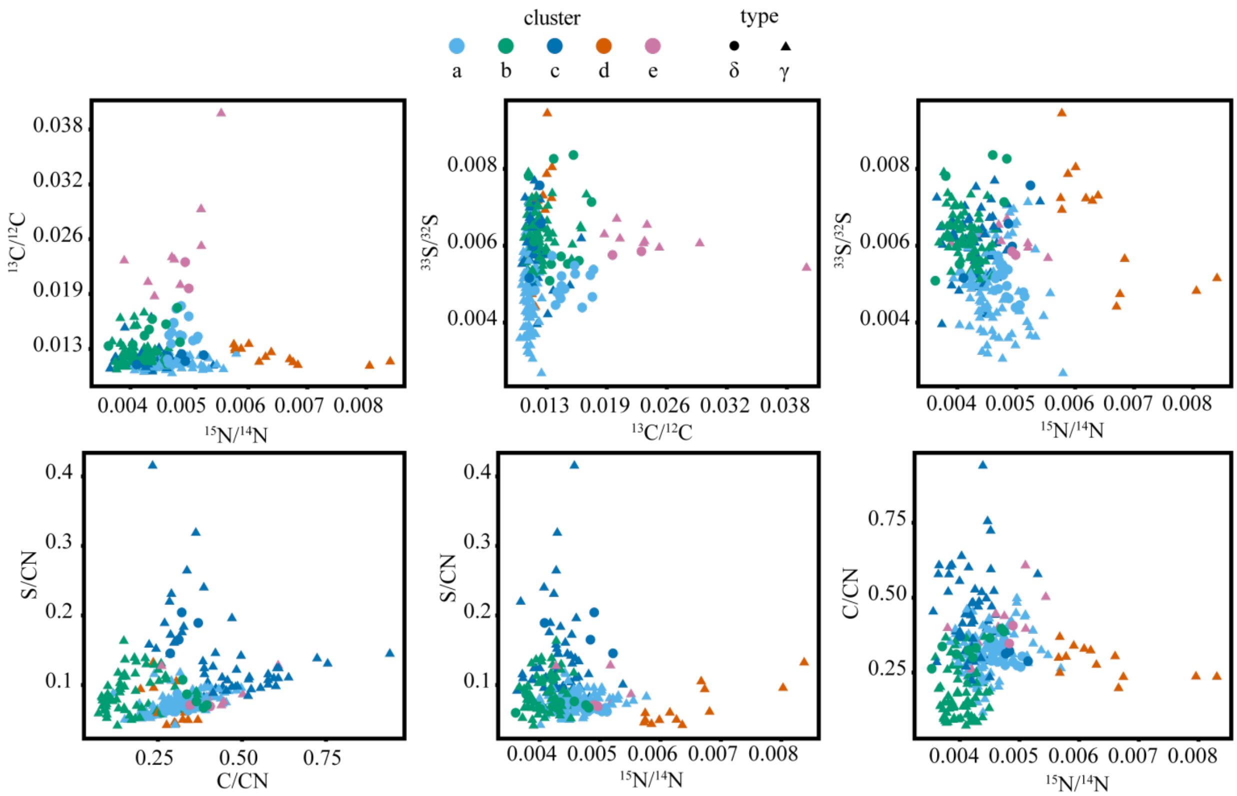

Figure S-2: Isotope and element ratio properties of the five clusters for the killed control sample showing minimal uptake of the ^{13}C and ^{15}N label in the killed control and a subset of ROIs with higher S/CN. All ROIs plot on a reduced scale for $^{13}C/^{12}C$, $^{15}N/^{14}N$ and $^{32}S/^{33}S$ as compared to

the live, labeled experiments. The killed control did not result in the affiliation of isotope phenotypes from clusters ‘a’ – ‘e’ with Gammaproteobacteria (‘g’) or Deltaproteobacteria (‘d’) as observed in the labeled substrate incubations.

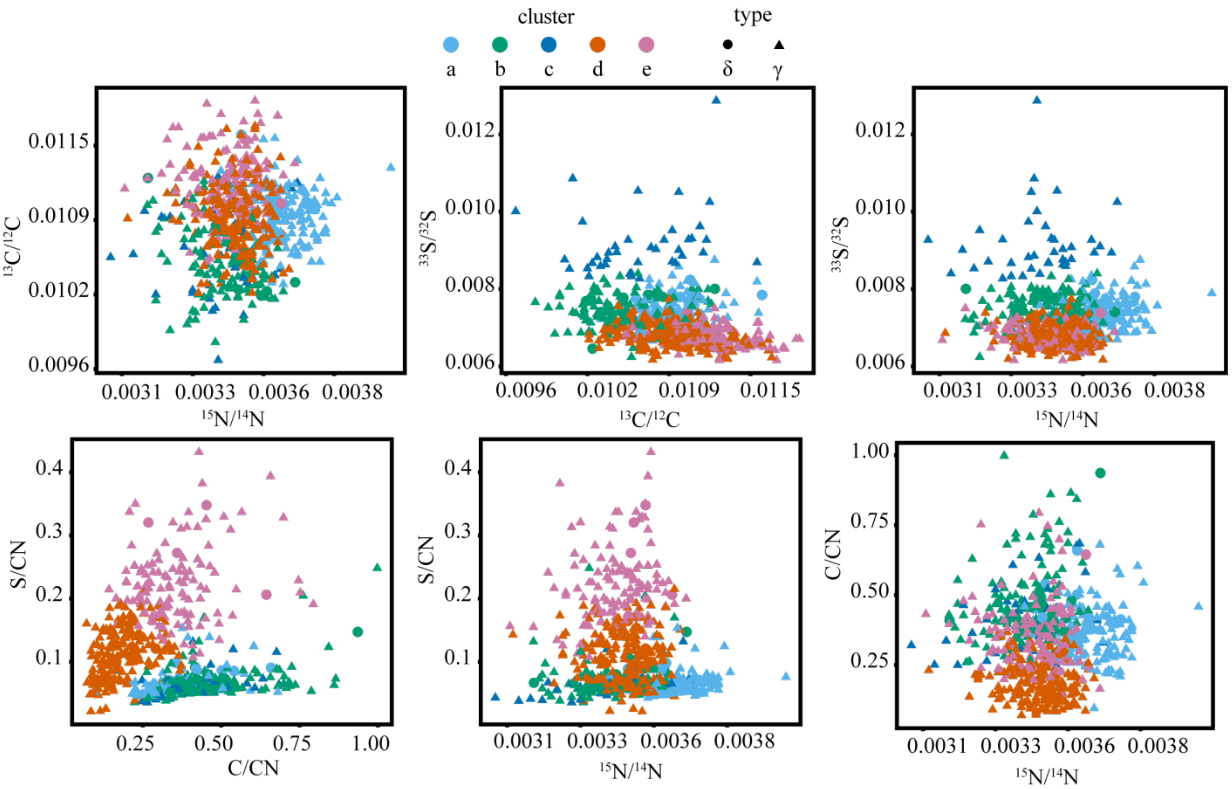

Figure S-3: Isotope and element ratio properties of the five clusters for the unlabeled control sample. All ROIs plot within one standard deviation of the calculated natural abundance value resulting in a reduced scale for $^{13}C/^{12}C$, $^{15}N/^{14}N$ and $^{32}S/^{33}S$ ratios as compared to the live, labeled experiments. The unlabeled control did not result in the affiliation of isotope phenotypes from clusters ‘a’ – ‘e’ with Gammaproteobacteria (‘g’) or Deltaproteobacteria (‘d’) as observed in the labeled substrate incubations.

2 days

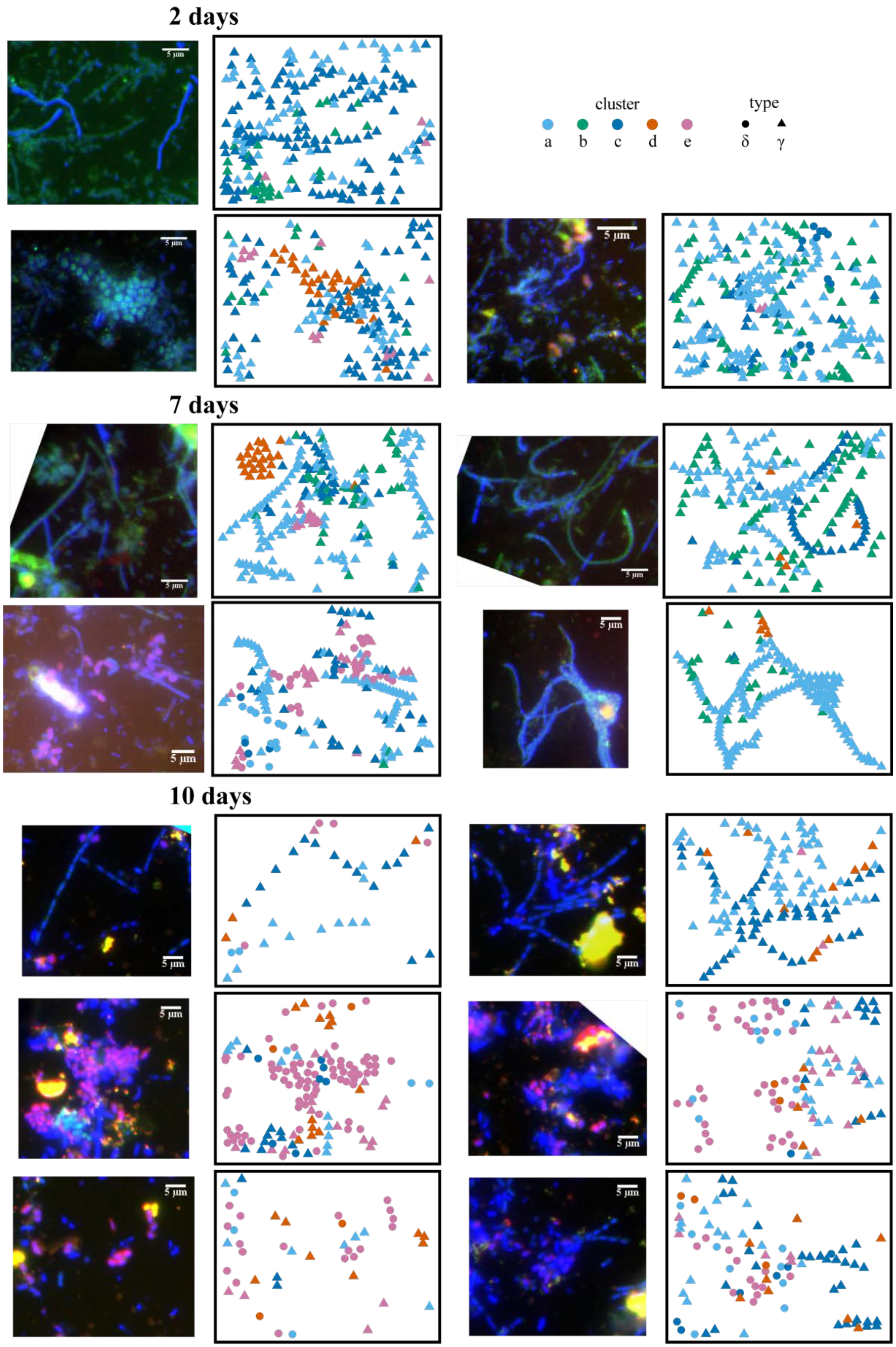

Figure S-4: Comparison of FISH and NanoSIMS cluster analysis for multiple images from 2, 7 and 10 days of incubation. FISH experiments (1st and 3rd panels) were performed with probes for

Deltaproteobacteria (purple) and Gammaproteobacteria (green). Clusters ‘a’, ‘b’ and ‘c’ are primarily affiliated with filamentous Gammaproteobacteria. Cluster ‘d’ is primarily affiliated with clusters of coccoid Gammaproteobacteria. Cluster ‘e’ is primarily affiliated with Deltaproteobacteria.
